# Probiotic Bifunctionality of *Bacillus subtilis*—Rescuing Lactic Acid Bacteria from Desiccation and Antagonizing Pathogenic *Staphylococcus aureus*

**DOI:** 10.3390/microorganisms7100407

**Published:** 2019-09-29

**Authors:** Hadar Kimelman, Moshe Shemesh

**Affiliations:** 1 Department of Food Sciences, Institute for Postharvest Technology and Food Sciences, Agricultural Research Organization (ARO), Volcani Center, Rishon LeZion 7528809, Israel; hadar.kimelman@mail.huji.ac.il; 2 Institute of Dental Sciences, Faculty of Dental Medicine, Hebrew University-Hadassah, Jerusalem 9112102, Israel

**Keywords:** beneficial biofilm, bio-coating, *B. subtilis*, probiotics, extracellular matrix, pathogen elimination

## Abstract

Live probiotic bacteria obtained with food are thought to have beneficial effects on a mammalian host, including their ability to reduce intestinal colonization by pathogens. To ensure the beneficial effects, the probiotic cells must survive processing and storage of food, its passage through the upper gastrointestinal tract (GIT), and subsequent chemical ingestion processes until they reach their target organ. However, there is considerable loss of viability of the probiotic bacteria during the drying process, in the acidic conditions of the stomach, and in the high bile concentration in the small intestine. *Bacillus subtilis*, a spore-forming probiotic bacterium, can effectively maintain a favorable balance of microflora in the GIT. *B. subtilis* produces a protective extracellular matrix (ECM), which is shared with other probiotic bacteria; thus, it was suggested that this ECM could potentially protect an entire community of probiotic cells against unfavorable environmental conditions. Consequently, a biofilm-based bio-coating system was developed that would enable a mutual growth of *B. subtilis* with different lactic acid bacteria (LAB) through increasing the ECM production. Results of the study demonstrate a significant increase in the survivability of the bio-coated LAB cells during the desiccation process and passage through the acidic environment. Thus, it provides evidence about the ability of *B. subtilis* in rescuing the desiccation-sensitive LAB, for instance, *Lactobacillus rhamnosus*, from complete eradication. Furthermore, this study demonstrates the antagonistic potential of the mutual probiotic system against pathogenic bacteria such as *Staphylococcus aureus*. The data show that the cells of *B. subtilis* possess robust anti-biofilm activity against *S. aureus* through activating the antimicrobial lipopeptide production pathway.

## 1. Introduction

Live probiotic microorganisms obtained often with food are thought to improve human health. Thus, probiotics are usually defined as live microbial cells that provide a health benefit to the host when administered in sufficient quantities [1]. Among most prominent probiotic microorganisms are Gram-positive lactic acid bacteria (LAB), which mainly belong to the *Lactobacillus* and *Bifidobacterium* genera [2]. The essential probiotic requirement in terms of the health benefits is a positive influence on the digestion and immune systems [3]. Moreover, probiotics also have a protective role, directly competing with pathogens through signaling interference [4], releasing antimicrobial substances [5] or metabolites such as acids [6,7,8]. Nevertheless, to exert its beneficial effects, any probiotic organism must survive, establish, and multiply in the host.

Probiotic bacteria are usually delivered as dried cultures, but the process used to prepare them may damage the cell’s structure, vitality, and functionality [9,10]. Drying processes involve the removal of a large amount of fluid from the cell, which affects the cellular structure and may, therefore, cause cell death [10,11]. Moreover, probiotic cells must survive shelf life and transit in the gastrointestinal tract, including acid stress in the stomach [11], as well as degradation by enzymes and bile salt in the intestine [12].

One of the main strategies of bacteria to deal with environmental stresses is the formation of a complex structure called a biofilm [13]. In most natural settings, bacteria do not grow as free-living cells but, instead, they form complex polymicrobial structures [14]. The biofilm structures contain less than 10% microorganisms, while the other 90% is the extracellular matrix (ECM) produced by the bacteria themselves. This ECM mainly consists of polysaccharides and other macromolecules such as proteins, enzymes, surfactants, DNA, and lipids [15]. Thus, the biofilm structure is capable of resisting extreme environmental conditions such as transit through the gastrointestinal tract or desiccation [16]. The ECM creates a microenvironment, which might lead to enhanced survival during desiccation [17]. Apparently, hygroscopic polysaccharides are thought to promote biofilm fluidity and resistance to desiccation [18].

*Bacillus subtilis*, a spore-forming nonpathogenic Gram-positive bacterium, is commonly found in the soil and the gastrointestinal tract (GIT) of some mammals [19]. This bacterium can effectively maintain a favorable balance of microflora in the GIT of the mammalian host [20]. As one of the physiological hallmarks, *B. subtilis* can form a robust biofilm through activation of a dedicated signaling pathway to coordinate expression of genes encoding the ECM [21,22]. Its ECM relies mainly on exopolysaccharides (EPS) synthesized by the *epsA-O* operon and amyloid fibers encoded by the *tasA* located in the *tapA–sipW–tasA* operon [23].

According to recent studies, the use of *Bacillus* species and especially *B. subtilis* as probiotics gained vast interest. Thus, *Bacillus* species were reported to be effective in preventing respiratory infections and gastrointestinal disorders, and overcoming symptoms associated with irritable bowel syndrome [24,25]. However, the mechanism(s) via which *Bacillus* species act as probiotics remains unclear. It appears that the presence of *B. subtilis* helps to maintain a favorable balanced microbiota in the gut and enhances probiotic LAB cell growth and viability [26]. It was also suggested that these probiotic properties are related to its ability to stimulate the immune system [24] and the production of antimicrobial substances [27,28], or even inducing signaling interference against pathogenic microorganisms [4].

Cells of *B. subtilis* produce a vast diversity of antimicrobial substances, amongst them, relatively well-characterized groups of lipopeptides, for instance, surfactins, iturins, and fengycins. These compounds have a wide variety of biological activities such as anti-bacterial, anti-fungal, anti-viral, and anti-tumor activities [29]. They can work in different mechanisms such as disrupting the structure of bacteria members, decreasing the surface and interfacial tension of biofilms [30], and inhibiting quorum sensing, which inhibits biofilm formation [4]. Furthermore, lipopeptides have an essential role in signaling for biofilm formation in *B. subtilis* [31,32].

A model system was recently developed, which enhances biofilm formation by *B. subtilis* through mutual growth with LAB [33]. This system seems to be beneficial for the protection of LAB during heat treatment and through passage in the gastrointestinal tract [33]. The current study presents a further development of the biofilm-based protective coating for probiotic cells via a process defined as a bio-coating. Furthermore, this study provides evidence for two different probiotic functionalities of *B. subtilis*: (i) protecting the LAB during their exposure to desiccation conditions and acid stress; (ii) showing potent anti-microbial activity against pathogenic bacteria such as *Staphylococcus aureus*.

## 2. Materials and Methods

### 2.1. Strains and Growth Conditions

Bacterial strains used in this study and their origins are summarized in Table S1 (Supplementary Materials). The lactic acid bacteria (LAB) were routinely grown in either MRS broth (Man, Rogosa & Sharpe) (Hy-labs, Rehovot, Israel) or MRS broth solidified using 1.5% agar (Difco, New-Jersy, USA). In addition, the wild-type (WT) strain NCIB3610 of *B. subtilis* and its derivatives (Table S1, Supplementary Materials) were regularly cultured in Lysogeny broth (LB containing: 10 g of tryptone, 5 g of yeast extract, and 5 g of NaCl per liter) (Difco) or LB solidified with 1.5% agar. Prior to generating starter cultures, LAB and *B. subtilis* cells were grown on the agar-solidified plates for 48 h or overnight, respectively, both at 37 °C. A starter culture of each strain was prepared using a single bacterial colony; the cells of LAB were inoculated into 5 mL of MRS broth overnight without agitation, while the cells of *B. subtilis* were inoculated into LB medium overnight at 30 °C, 150 rpm, until the cultures reached an OD_600_ of approximately 1.5. For co-culture experiments, the modified MRS (MMRS) medium (pH = 7) was used due to its biofilm-promoting capability and its suitability for co-culture cultivation of *B. subtilis* and LAB [33]. Thus, *B. subtilis* cells were mixed with an equal amount of the LAB cells to a final concentration of 10^8^ cells/mL of each strain within MMRS. The cells in mixed cultures were incubated aerobically at 37 °C at 50 rpm for 8 h [33]. Cells of *S. aureus* ATCC 25923 were regularly cultured in tryptic soy broth (TSB) (Difco) solidified with 1.5% agar overnight at 37 °C. A starter culture was prepared using a single bacterial colony inoculated into 10 mL of TSB medium overnight at 37 °C, 150 rpm.

### 2.2. Visualizing Biofilm-Forming Cells Using Confocal Laser Scanning Microscopy (CLSM)

Unlabeled cells of LAB and the CFP-tagged *B. subtilis* cells (YC189) were grown in co-culture into MMRS broth as described above. Cell suspensions of each bacterium grown as mono-species culture served as control samples. One milliliter of each culture was collected and centrifuged at 5000 rpm for 2 min. After removing supernatant, the cells were washed with 1 mL of DW (distilled water) and then the following centrifugation (at 5000 rpm for 2 min) re-suspended in 100 µL of DW. A suspension of 5 µL from each sample was placed on a microscopy glass slide and visualized in a transmitted light microscope using Nomarski differential interference contrast (DIC) and 458-nm laser for CFP excitation (Leica, Wetzler, Germany).

### 2.3. Growth Curve Analysis of Lab During Growth in Co-Culture

Initially, *B. subtilis* and LAB cells were grown overnight in either LB or MRS as described above. Afterward, the cells were introduced into the MMRS medium, and the co-culture was incubated for 8 h at 37 °C at 150 rpm. Mono-species cultures of LAB and *B. subtilis* were used as control. Every 2 h, 1 mL of each sample was collected for quantification of bacteria by the colony forming unit (CFU) counting method on either MRS (for LAB) or LB (for *B. subtilis*) agar plates. The plates were incubated aerobically at 37 °C for either 48 h (in case of LAB) or 24 h (for *B. subtilis*).

### 2.4. Analysis of Survival Rates Following Desiccation Treatment

The co-culture samples generated as described above were grown for 8 h aerobically at 37 °C and 50 rpm. The LAB cells grown as a mono-culture were used as a control. One milliliter of each sample was harvested by centrifugation at 4000× *g* for 10 min, washed once with DW, and 50 µL of the sample was placed into wells of a 96-well polystyrene plate (Greiner Bio-One, Monroe, North Carolina, USA). The plate was left open in a dry cabinet (MRC, Holon, Israel), at 40% relative humidity and 25 °C for 20 to 40 h. The MRS agar plates were used to determine the cell counts before drying. For analysis of the viable cell counts following desiccation, 100 µL of DW was added to each well, and the plates were incubated for 5 min at room temperature, before resuspension of the samples by pipetting. Bacterial suspensions were serially diluted and underwent CFU counting on MRS agar plates. The CFU counts were recorded following incubation for 48 h at 37 °C.

### 2.5. Visualizing Co-Culture Biofilm Following Desiccation Treatment Using Scanning Electron Microscopy (SEM)

The co-culture and mono-culture samples were generated as described above. One milliliter of each sample was harvested by centrifugation (at 4000× *g* for 10 min) and washed once with DW. Then, 5 µL of suspension from each sample was placed on polylysine-coated glass slides and left open in the dry cabinet at 40% relative humidity and at 25 °C for 20 h. Before analysis in the SEM, the slides were coated by gold/palladium coating (20:80), at 12 mA voltage and 1 nm thickness.

### 2.6. Analysis of Survival Rates Following Freeze-Drying

The co-culture and mono-culture samples were generated as described above. Afterward, the samples were harvested by centrifugation at 4000× *g* for 10 min. One milliliter of each sample was washed and resuspended with DW; the suspensions were then mixed with an equal volume of skim milk (10%), to optimize the cell viability [34]. The samples were placed in a −80 °C freezer for 48 h and subsequently freeze-dried in lyophilizer (Ilshin, Hialeah, FL, USA) for 24 h. The survival rates following the freeze-drying are expressed as the number of CFUs/mL. The cell survivability (following freeze-drying) was studied during incubation of the samples in pH 2 (with 1 M hydrochloric acid) at 37 °C, for either 1 h or 3 h, using the CFU counts.

### 2.7. Analysis of Survival Rates Following Transition within In Vitro Digestion System

To analyze the survivability of the LAB during the passage in the gastrointestinal tract, the freeze-dried samples were resuspended in 5 mL of DW. Afterward, the samples were monitored for four hours through an in vitro digestion system using the method described previously [35].

### 2.8. Determining the Effect of Conditioning Supernatant (CSN) on S. aureus Biofilm Formation

Cells of *B. subtilis* and *Lactobacillus plantarum* were grown either in monoculture or co-culture using MMRS medium as described above for 24 h; *B. subtilis* cells were also grown in LB medium. Cultures were then centrifuged at 13,000 rpm for 5 min, and the supernatants were sterilized by passing through a 0.45-µm filter (Merck, Rockland, MA, USA). For analysis of the CSN effect on biofilm formation by pathogenic bacteria, the *S. aureus* starter culture was generated through its growth into 10 mL of TSB medium overnight at 37 °C and 150 rpm. The, *S. aureus* biofilm was grown into 1 mL TSB medium (within a 24-well culture plate) supplemented by the CSN (10% v/v) harvested from the above probiotic cultures. The TSB medium without supernatant was used as control. The plate was incubated for 24 h at 37 °C.

### 2.9. Biofilm Quantitation Assay

Crystal violet staining was performed similarly as described previously [34]. Briefly, following 24 h of incubation at 37 °C, unattached cells were removed by washing the well plates two times using DW. Then, 1% crystal violet (CV) solution was added to the wells. Following 2 min of incubation, the excess CV was removed by washing with DW. Afterward, the fixed CV was released by 33% acetic acid washing. Then, 100 μL of each sample was transferred to a new well plate for the absorbance detection at 570 nm. To confirm the CV results, the CFU quantitation was performed for surface-attached cells. Following 24 h of incubation at 37 °C, unattached cells were removed by washing the well plate two times using DW. Then, 1 mL of DW was added to each well, and the cells attached to the surface were scratched out using sterilized swab; the bacterial suspensions were serially diluted and underwent CFU counting on LB agar plates.

### 2.10. Effect of Cell-Free Culture Supernatant on S. aureus Growth

The, *S. aureus* starter culture was prepared into 10 mL of TSB medium overnight at 37 °C and 150 rpm. Then, 150 µL of the generated bacterial suspension was introduced into 15 mL of fresh TSB medium. For the antibacterial test, the *S. aureus* suspension was supplemented by the cell-free supernatant prepared as described above. The samples were incubated for 8 h at 37 °C at 150 rpm and subjected to OD_600_ measurements every 1 h.

### 2.11. Confocal Laser Scan Microscopy (CLSM) Analysis

The, *S. aureus* biofilm was grown in a confocal microscopy dish (glass-bottom dish) (Bar-Naor, Petach-Tikwa, Israel) with or without supplementation of the supernatant at the same conditions as described above. After 24 h of incubation at 37 °C, the surface-unattached cells were removed by washing the dish two times using DW. Next, the biofilm cells were stained using FilmTracer LIVE/DEAD Biofilm Viability Kit (Molecular Probes, Eugene, OR, USA) and incubated for 30 min in room temperature without exposure to light. Then, the stain was washed away and analyzed by confocal laser microscopy (CLSM) (Olympus, Hamburg, Germany). Fluorescence emission from the stained samples was measured with an SP8 CLSM (Leica) equipped with 488- and 552-nm lasers.

### 2.12. Real-Time PCR

To further analyze the potential antimicrobial effect of *B. subtilis* grown in MMRS medium, we tested the expression of genes that could be affected during mitigating biofilm formation by *S. aureus*. A starter culture of *B. subtilis* was prepared during overnight growth at 30 °C, 150 rpm, in LB medium. For generating the antimicrobial substance producing a suspension of *B. subtilis*, a portion of starter culture was introduced (by 1:100 ratio) into either MMRS or LB medium (as a control medium for a low antimicrobial substance production). The samples were incubated for 6 h at 37 °C, 50 rpm. Next, 2 mL from each sample was collected and centrifuged at 5000× *g* for 10 min. The RNA was harvested using the RNAeasy kit (QIAGEN, Hilden, Germany) following the manufacturer’s protocol. The RNA concentration was measured by means of a Nanodrop 2000 spectrophotometer (ThermoFisher Scientific, Waltham, MA, USA). A complementary DNA (cDNA) was synthesized from 1 μg of RNA in a reverse transcription reaction using a qScript cDNA Synthesis Kit (Quantabio, Beverly, MA, USA) according to the manufacturer’s instructions. All cDNA samples were stored at −20 °C. The RT-PCR reactions (final volume = 20.0 μL) consisted of 2 μL of cDNA template, 10 μL of fast SYBR green master mix, 1 μL of suspension of each primer, and 7 μL of RNase free water. Forward and reverse PCR primers (Table S2, Supplementary Materials) were designed using the Primer express software and were synthesized by Hylabs (Rehovot, Israel). DNA was amplified with the Applied Biosystems StepOne™ Real-Time PCR System (Life technologies, Foster, CA, USA) under the following PCR conditions: initial denaturation for 2 min at 95 °C and subsequent 40 PCR cycles (95 °C for 3 s, 60 °C for 30 s, and 95 °C for 15 s). The RNA samples without reverse transcriptase were used as negative control, to confirm that there was no DNA contamination in the RNA samples. The expression levels of the tested genes (*fenA, srfA*) were normalized using the 16S ribosomal RNA (rRNA) and *rpoB* genes as the endogenous controls (Table S2, Supplementary Materials).

### 2.13. Statistical Analysis

The results were subjected to either Student’s *t*-test or one-way analysis of variance at a significance level of *p* < 0.05, to compare the control and tested samples. The results are based on three biological repeats performed in duplicates.

## 3. Results

### 3.1. Formation of Mutual Probiotic Biofilm of B. subtilis with Lactic Acid Bacteria (LAB)

**Figure 1:**
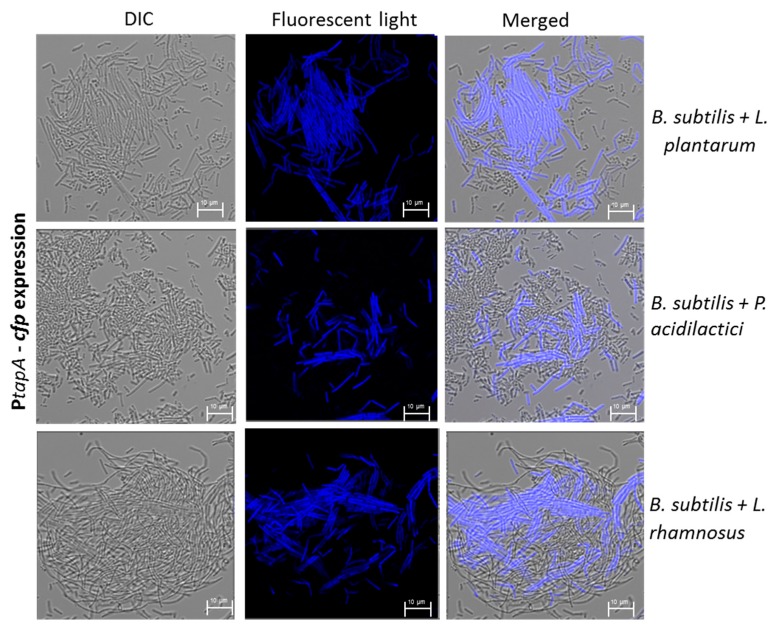
Formation of dual-species biofilm bundles of *B. subtilis* with LAB. Bacterial biofilms were generated during co-culture growth of *B. subtilis* cells with either *Lactobacillus plantarum*, *Pediococcus acidilactici*, or *L. rhamnosus* cells in modified MRS (MMRS) medium at 37 °C for 8 h. The biofilm samples were prepared as described in Section 2 and analyzed using a confocal laser scanning microscope (CSLM, Leica, Germany). *B. subtilis* cells express CFP under the control of the *tapA* operon, which is responsible for the matrix production. LAB cells are not stained. Scale bar = 10 µm.

The starting point of this investigation was generating the dual-species biofilms for the different LAB strains together with robust ECM-producing bacterium *B. subtilis*. Thus, the bacterial cells were incubated in the biofilm-promoting MMRS medium, which promotes increased biofilm formation by *B. subtilis* through the KinD-Spo0A pathway [33]. To visualize the mutual biofilms, a transcriptional fusion of the *tapA* promoter to the *cfp* gene (encoding cyan fluorescent protein) was used [35]. The observed upregulation in the CFP expression, during the mutual growth of *B. subtilis* with three different species of the probiotic LAB, indicates that the *tapA* operon was activated and there that there was notable matrix production by *B. subtilis* (Figure 1). This finding was quite noticeable following a comparison of morphological changes that occurred during LAB growth in the presence of *B. subtilis* (Figure S1, Supplementary Materials). In this regard, the LAB cells could not form any biofilm bundles during their growth as a mono-species culture (Figure S1, Supplementary Materials), whereas a notable incorporation of those cells was observed into biofilm bundles produces by *B. subtilis* cells (Figure 1). 

**Figure 2:**
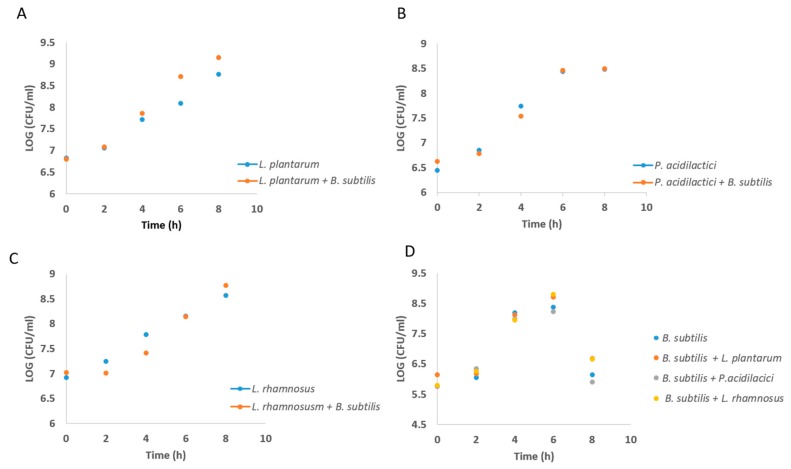
Co-culture growth of *B. subtilis* with the LAB bacteria does not influence growth rate. Growth curve analysis of LAB cells in presence or absence of *B. subtilis*. The blue line represents the growth rate of a single-species culture of LAB, whereas the orange line represents the growth rate of (**A**) *L. plantarum,* (**B**) *P. acidilactici*, and (**C**) *L. rhamnosus* in co-culture with *B. subtilis*. (**D**) Growth curve of *B. subtilis* cells in the presence of LAB species compared to single culture. The cells were analyzed during growth for 8 h in 37 °C, 150 rpm.

To confirm that there are no antagonistic interactions between the LAB and *B. subtilis* cells, the bacterial growth was analyzed in this mutual growth system. Consequently, there was no significant inhibition in either of the bacterial species following their mutual growth (Figure 2), meaning that the LAB and *B. subtilis* cells can grow together without interference through generating the mutual probiotic biofilm. In addition, the growth in dual-species biofilm did not change the medium acidification rate by the LAB cells (Figure S2, Supplementary Materials).

### 3.2. Growth in Mutual Biofilm Increases the Survivability of the LAB during Desiccation

**Figure 3:**
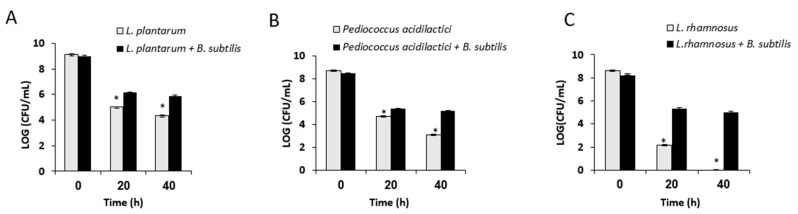
Growth in dual-species biofilm increases survivability of LAB during exposure to drying process. Mono and dual-species cultures of *B. subtilis* and LAB cells were generated in MMRS medium during bacterial growth for 8 h in 37 °C, 50 rpm. Survival rates of the LAB cells (grown in presence or absence of biofilm forming *B. subtilis*) were determined based on CFU counts following desiccation conditions (40% relative humidity (RH)) for 20–40 h. Error bars represent standard deviation (SD). * *p* < 0.05 comparison of the control and tested samples. (**A**) *L. plantarum* survival rates, (**B**) *P. acidilactici* survival rates, and (**C**) *L. rhamnosus* survival rates.

It was hypothesized that the growth in the mutual biofilm system could provide a relative protection of LAB during the desiccation process, which might indicate about the relative improvement in survivability of the bio-coated cells through industrial processing and storage conditions. Therefore, the LAB cells grown in the mutual biofilm were exposed to desiccation conditions for either 20 or 40 h. The LAB cells grown in mono-species culture were used as a control sample. It was found that *L. plantarum* cells grown in mutual biofilm showed increased survival (relatively to mono-species culture) of around 1.12 log·CFU/mL and 1.52 log·CFU/mL, following 20 and 40 h of desiccation, respectively. Surprisingly, *L. rhamnosus* cells grown in the mutual biofilm demonstrated an even more significant increase in survivability, of around 3.12 log·CFU/mL, during 20 h of desiccation. Even more profoundly, the bio-coated cells of *L. rhamnosus* demonstrated around a five-log increase in their survivability after 40 h of desiccation. Concerning the cells of *P. acidilactici*, the bio-coated cells showed a relatively moderate increased survival following 20 and 40 h of desiccation of around 0.71 log·CFU/mL and 2.09 log·CFU/mL, respectively (Figure 3).

**Figure 4:**
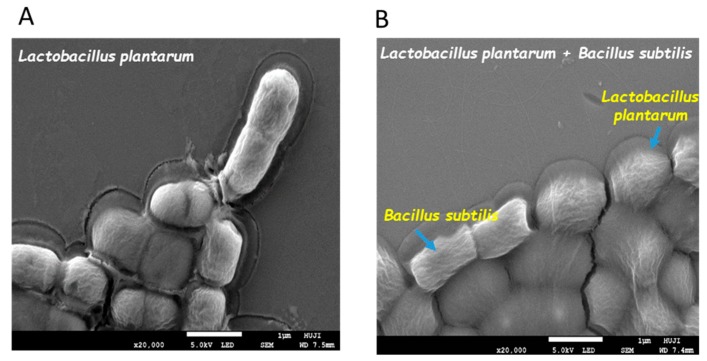
Bio-coating of *L. plantarum* cells by extracellular polymeric substance(s) produced by *B. subtilis*. Mono and dual-species cultures of *B. subtilis* and *L. plantarum* cells were generated in MMRS medium during bacterial growth for 8 h in 37 °C, 50 rpm. (**A**) SEM images of the mono-species culture of *L. plantarum* and (**B**) dual-species culture of *L. plantarum* with *B. subtilis* following desiccation conditions (40% RH) for 20 h. Images were taken at a magnification of 20,000× with a JEOL, JSM 7800F, Japan.

To reinforce our assumption about the ECM protection of the LAB cells, the dual- and mono-species biofilms were visualized using SEM imaging, after desiccation treatment. It was found that *L. plantarum* cells grown in the presence of *B. subtilis* were surrounded with the coating substance(s), which could be interpreted as the biofilm matrix (Figure 4).

### 3.3. Growth in Mutual Biofilm Increases the Survivability of the LAB during Acid Stress Following Freeze-Drying

**Figure 5:**
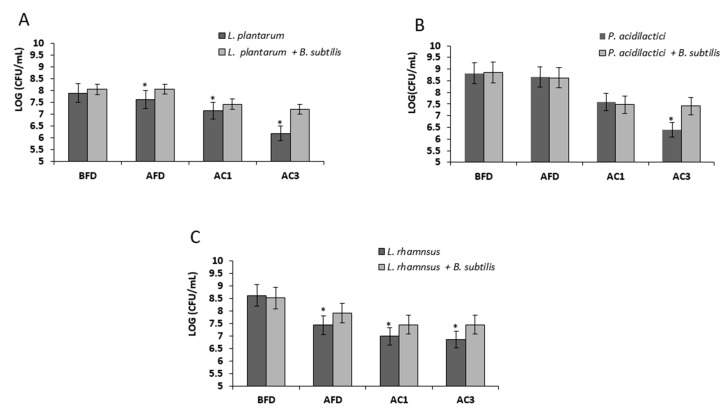
Bio-coating increases the survivability of the LAB during acid stress following freeze-drying. Mono- and dual-species cultures of *B. subtilis* and the LAB cells were generated in MMRS medium during bacterial growth for 8 h in 37 °C, 50 rpm. Survival rates of LAB cells were determined based on CFU counts following freeze-drying and an exposure to low pH (HCl 1 M, pH = 2) for 1–3 h. * *p* < 0.05 for comparison of the control and tested samples. Error bars represent standard deviation (SD). BFD—before freeze-drying; AFD—after freeze-drying; AC1—freeze-dried and exposed for 1 h to acid conditions; AC3—freeze-dried and exposed for 3 h to acid conditions. The survival rates are shown for (**A**) *L. plantarum*, (**B**) *P. acidilactici*, and (**C**) *L. rhamnosus.*

It was further investigated whether the mutual biofilm could also protect the cells of LAB during freeze-drying, which is considered as the most common technique for drying and storage of probiotic bacteria for a long time [10,36]. Since, after consumption, the LAB cells are usually exposed to additional stress (acidic stress during the passage in the gastrointestinal tract), the survivability of the bio-coated cells during their exposure to acid stress was tested, following freeze-drying. To mimic the acid stress conditions, the bio-coated cells were freeze-lyophilized and exposed to pH 2. It was found that the bio-coated *L. plantrum* cells showed increased survivability of around 0.45 log·CFU/mL, compared to the control, following freeze-drying (Figure 5). In the case of the bio-coated *L. rhamnosus* cells, an increase of about 0.49 log·CFU/mL was observed, while, in the case of *P. acidilactici*, there was no significant change in the number of viable cells following freeze-drying (Figure 5). Concerning acid stress tolerance, the freeze-dried LAB cells were exposed to low pH (pH 2) for 1–3 h. As shown in Figure 5, the encapsulated LAB consistently demonstrated increased survival (up to around a one-log increase) following their exposure to this stress, especially after three hours of exposure to the low pH.

### 3.4. Bio-Coating Retains the LAB Survivability during In Vitro Gastrointestinal Digestion Following Freeze-Drying

**Figure 6:**
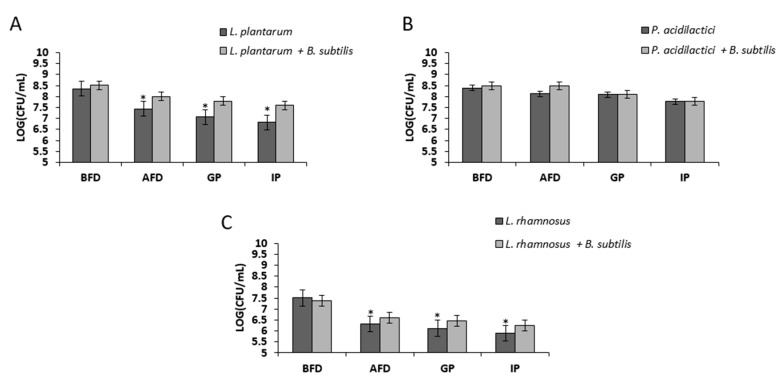
Bio-coating maintains LAB survivability during gastrointestinal digestion in vitro following freeze-drying. Mono- and dual-species cultures of *B. subtilis* and LAB cells were generated in MMRS medium during bacterial growth for 8 h in 37 °C, 50 rpm. Survival rates of LAB cells were determined based on CFU counts following freeze-drying and during gastro-intestinal digestion in vitro. * *p* < 0.05 for comparison of the control and tested samples. Error bars represent standard deviation (SD). BFD—before freeze-drying; AFD—after freeze-drying; GP—gastric phase; IP—intestinal phase. The survival rates are shown for (**A**) *L. plantarum*, (**B**) *P. acidilactici*, and (**C**) *L. rhamnosus*.

It was further tested whether bio-coating could also provide protection of the LAB cells during passage through an in vitro GIT system. This unique system included two phases: a gastric phase characterized by low pH and stomach proteolytic enzymes and, the intestinal phase, characterized by neutral pH, intestinal proteolytic enzymes, and bile salts. The bio-coated LAB cells were freeze-lyophilized and tested through this system. It was found that the bio-coated cells of *L. plantarum* and *L. rhamnosus* showed increased survivability of around 0.5 log following freeze-drying, which remained at the augmented level during the transition of those cells through the GIT system (Figure 6). Nonetheless, in the case of *P. acidilactici*, there was no significant increase in the survivability of the cells in the tested conditions.

### 3.5. Antagonistic Effect of Probiotic Cells against the Biofilm-Forming, S. aureus

**Figure 7:**
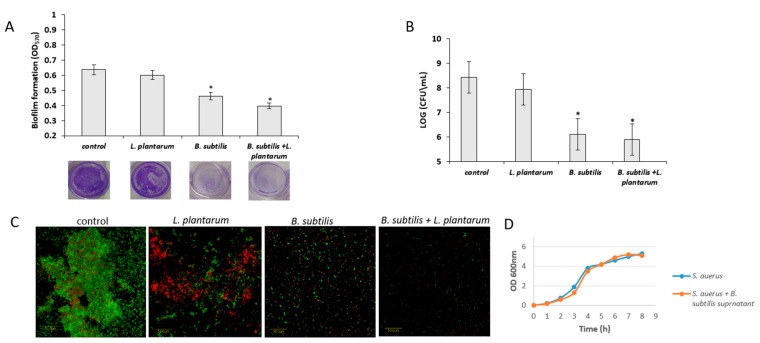
Conditioning supernatant (CNS) of the *B. subtilis* culture inhibits biofilm formation by *Staphylococcus aureus*. *S. aureus* biofilm formation was determined after growth in TSB medium with 10% CNS at 37 °C for 24 h. (**A**) *S. aureus* biofilm formation quantification by crystal violet method. (**B**) Quantification of live bacteria attached to surface using CFU method. * *p* < 0.05 for comparison of the control and tested samples. Error bars represent standard deviation (SD). (**C**) Confocal laser microscopy (CLSM) images of *S. aureus* biofilms formed onto polystyrene surfaces containing 10% CNS. Live cells (SYTO-9, green) and dead cells (propidium iodide, red). Scale bar = 50 µm. (**D**) *S. aureus* growth curve in TSB medium supplemented by the 10% CNS generated from the *B. subtilis* growth medium.

Next, it was hypothesized that the probiotic cells (from the mutual biofilm) could antagonize pathogenic bacteria, for instance, *S. aureus*, which is known as a robust biofilm-forming bacterium, especially a submerged type of biofilm. It was consequently found that the conditioning supernatant (CSN) obtained during the growth of the probiotic cells strongly inhibited biofilm formation by *S. aureus* (Figure 7A). This result indicates that there might be an induction in producing an antimicrobial substance(s) during the generation of the mutual biofilm. Interestingly, it seems that a major impact of this inhibitory effect was related to *B. subtilis* cells, although there was a modest contribution by the cells of *L. plantarum*. A further quantitation of the surface-adhered cells confirmed a potent inhibitory effect of the CSN against biofilm formation by pathogenic *S. aureus* (Figure 7B). Moreover, the microscopic visualization of the augmented biofilm phenotypes confirmed once again the anti-staphylococcal properties of the CSN (Figure 7C). Importantly, it was further confirmed that the CSN did not cause growth inhibition of *S. aureus* (Figure 7D), which indicates the biofilm-specific mode of action of CNS against this pathogenic bacterium.

**Figure 8:**
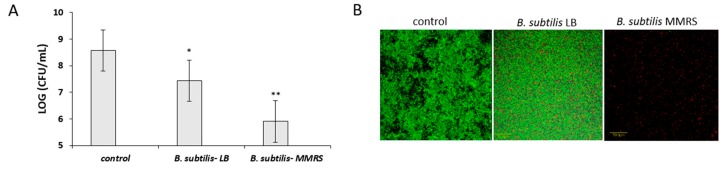
Type of growth medium governs the inhibitory effect on the *S. aureus* biofilm formation. (**A**) quantification of the *S. aureus* cells attached to the surface using CFU method, and (**B**) the CLSM imaging of the *S. aureus* biofilm formation in the presence or absence of the CSN, following growth in TSB medium with 10% supernatant at 37 °C for 24 h. * *p* < 0.05 for comparison of the CSN from LB to control; ** *p* < 0.05 for comparison of CSN from MMRS vs. LB. Error bars represent standard deviation (SD). Live cells are stained green (SYTO-9) and dead cells are stained red (propidium iodide). Scale bar = 50 µm.

It was further hypothesized that the growth media would affect the ability of the CSN to inhibit *S. aureus* biofilm formation. Therefore, the antibiofilm activity of the CNS produced by *B. subtilis* cells in the MMRS medium was compared with that produced in LB medium. Apparently, growth of the *Bacillus* cells in the MMRS induced the antibiofilm effect of the CSN (Figure 8). Thus, a significantly higher inhibition on *S. aureus* biofilm formation was found by the CSN from the MMRS medium compared to that produced in the LB medium. Accordingly, the CSN from MMRS medium showed around a three-log reduction in the *S. aureus* adherence onto the surface compared to that from LB (Figure 8A). The inhibitory effect of the CSN was further confirmed microscopically by testing a submerged biofilm of *S. aureus* cells using live–dead staining (Figure 8B).

**Figure 9:**
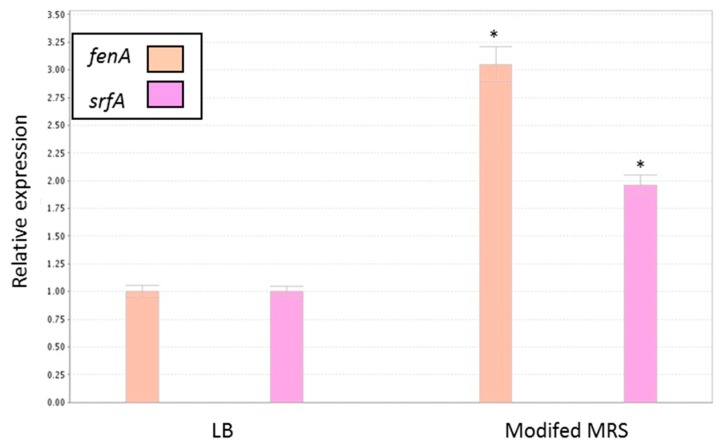
Relative expression of *B. subtilis* genes related to antagonistic activity during growth in the different media. The real-time (RT)-PCR analysis was performed for quantitation of *fenA* and *srfA* gene expression in *B. subtilis* cells grown in either LB or MMRS medium as described in the Methods. * *p* < 0.05 compared to control. Error bars represent standard deviation (SD).

According to recent findings, *B. subtilis* could affect *S. aureus* biofilm formation via signaling interference [4]. It was, thus, hypothesized that the production of either fengycins [4] or surfactin [37] could explain the antibiofilm activity of the CSN produced by *B. subtilis.* Both factors are produced by *B. subtilis*, and they could affect the *S. aureus* cells through interfering with inter- or intra-cellular signaling. It was subsequently found that expression of the genes encoding for these factors by *B. subtilis* was notably upregulated in the MMRS compered to LB medium (three- and two-fold induction in the expressions of *fenA* and *srfA*, respectively) (Figure 9). This result suggests the involvement of the regulatory pathways associated with those genes in the observed antibiofilm phenotype.

## 4. Discussion

The importance of healthy commensal microbiota for the mammalian host is evident; thus, there is widespread use of probiotics for preventing and treating various health problems in humans, as well as in animals. Nonetheless, maximizing the survivability of probiotic cells during their formulation, as well delivery, remains a significant challenge. Probiotic bacteria are supposed to go through a long route starting with processing, through shelf life and the passage through the GIT, which includes dealing with extreme conditions [38]. Since these processes may affect cell survivability, an effective way(s) of delivering probiotic bacteria to the mammalian host would be highly useful.

It is now well established that biofilm formation represents one of the most favorable microbial lifestyles within often challenging natural environments [39]. The biofilm provides bacterial cells protection against challenging environmental conditions such as changes in shear forces, extreme temperatures, desiccation, extreme pH, and antimicrobial agents [40,41,42]. It was, therefore, proposed to generate a protective bio-coating system for probiotic cells for a possibility of a more efficient delivery to the mammalian host. The most appropriate candidate for this mission appeared to be the robust biofilm-forming *B. subtilis*, since it naturally colonizes the mammalian gut and is considered to be harmless to mammals including humans [19,20]. It was confirmed that there are no antagonistic interactions during the generation of this complex multispecies system; thus, no antagonism was observed between *B. subtilis* cells and LAB, or during the formation of symbiotic biofilm bundles through inducing the expression of *tapA* operon (involved in the matrix production) by *B. subtilis*. It should be emphasized that the tested LAB strains belong to different genera with a different origin. Nonetheless, it was possible to generate cooperating and protected multispecies biofilms, which indicates the feasibility of using this bio-coating system for a wide range of probiotic species.

As suggested throughout the study, the generated bio-coating system increased the LAB survivability during drying processes, which points to the feasibility of using this system for processing probiotic cells for further food or biotechnological applications. The drying process is commonly used as a means for storage and distribution, which lowers the expense and inconvenience of using a cool chain. Although water is essential for bacteria living and the drying processes damage cell structure and viability, the long-time preservation and retention of viability during storage is often enhanced by lowering the water activity [34]. The robust biofilm matrix, produced by *B. subtilis*, contains polysaccharides (PS) that presumably have an important role in protecting bacteria during drying processes. The PS could provide bacteria a hydrated microenvironment. Thus, through the drying process, the PS layers may serve as a barrier on the cell surface and prevent water removal [18,43]. In the case of *L. rhamnosus*, which could not survive the 40 h of desiccation, the bio-coating process enabled a very significant increase in survivability during the desiccation process. This finding highly suggests the possibility of protecting desiccation-sensitive probiotic cells using this bio-coating system.

Freeze-drying is the most common drying method for long-term preservation of microorganisms, in the microbiological industry, thanks to optimal protection of cell viability [36]. Usually, before the freeze-drying process, protective agents like skim milk, sucrose, or other sugar types are added to the drying medium to prevent cell damage during the drying process and storage of freeze-dried cells [44]. Some studies showed that biofilm PS can also be used as a protective agent [43,45], which is in agreement with the findings of current study. We observed higher survivability during freeze-drying for *L. plantarum* and *L. rhamnosus* cells following their growth through the bio-coating system. However, we did not observe a significant increase in survival rates for *P. acidilactici* following freeze-drying. One of the possible explanations for this result could be related to the possible resistance of this environmental isolate to desiccation stress due to its adaptation to the udder environment (from where it was isolated).

In addition to the protective capability, *B. subtilis* demonstrated potent antimicrobial activity against pathogenic *S. aureus*. This result was not surprising since *B. subtilis* was recently explored for its probiotic functionality on many levels. It was shown that *B. subtilis* could stimulate an immune response in humans, as well as maintain a favorable balanced microbiota, and decrease infection and diarrhea via the synthesis of antimicrobial agents [46]. Production of antimicrobial agents is one of the antagonistic properties of probiotic bacteria, and indeed *B. subtilis* produces a wide diversity of substances, which influence a broad spectrum of pathogens via different mechanisms [27]. Several studies suggested either the growth inhibition or depression of *Staphylococcal* virulence by the antagonistic activity of *B. subtilis* [4,47]. In agreement with the literature, the current study showed that most of the antimicrobial activity of the multispecies biofilm system was due to substances produced by *B. subtilis*. Importantly, this activity was specific to the mitigation of biofilm formation by *S. aureus* rather than inhibition of its growth. Notably, the relatively modest effect of the CSN derived from *L. plantarum* on biofilm formation by *S. aureus* cells might still have an important role in mitigating this problematic pathogen. The synergistic activity of substances produced by different probiotic species might provide a further antimicrobial effect against such persistent pathogens.

Findings of this study further indicated that the inhibitory effect of the CSN is associated with the production of secondary metabolites, for instance, lipopeptides by *B. subtilis*. In this regard, the inhibitory effect could be related to their chemical structure [30] or to their ability to inhibit quorum sensing [4]. Lipopeptides produced by *B. subtilis* function firstly as quorum-sensing interrupters (fengycins), by inhibiting the quorum-sensing regulatory system [4], and surfactin, by regulating the autoinducer-2 (AI-2) activity [38]. Thus, these lipopeptides could inhibit quorum sensing via a different mechanism. The other antimicrobial mode of action of lipopeptides is related to the similarity of their chemical structure to surface-active agents, which might impair the ability of cells to attach to the surface and form a biofilm structure [30]. According to the results presented in this study, it appears that the growth of *B. subtilis* in the MMRS medium triggers the production of antimicrobial lipopeptides. This finding is indeed conceivable since the MMRS medium notably induces biofilm formation in *B. subtilis* [33], which is highly related to the production of antimicrobial lipopeptides [32].

Taken together, the data shown in this study suggest of the robust probiotic functionality of *B. subtilis* (i) in protecting the probiotic LAB during their exposure to unfavorable environmental conditions, such as desiccation and acid stresses, and (ii) strong anti-biofilm activity against pathogenic bacteria such as *S. aureus*.

## Supplementary Materials

The following are available online at https://www.mdpi.com/2076-2607/7/10/407/s1: Figure S1: The CSLM images of LAB cells following their monoculture growth; Figure S2: Growth in dual-species biofilm does not change medium acidification rate by LAB cells; Table S1: Bacterial strains used in this study; Table S2: Primers used for RT-PCR analyses.
