# Supplementary material for: Probiotic Bifunctionality of Bacillus subtilis—Rescuing Lactic Acid Bacteria from Desiccation and Antagonizing Pathogenic Staphylococcus aureus
Source: Microorganisms. 2019 Sep 29;7(10):407. doi: 10.3390/microorganisms7100407 (PMC6843919; doi:10.3390/microorganisms7100407)
Supplement: Supplementary file 1 [file microorganisms-07-00407-s001.pdf]

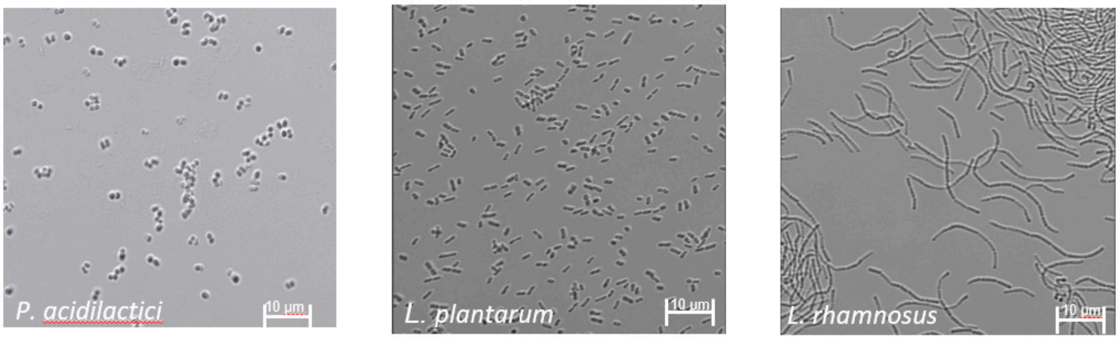

Figure S1. The CSLM **images of LAB cells following their monoculture growth.** The cells of *P. acidilactici L. plantarum*, or *L. rhamnosus* were grown in MMRS medium at 37 °C for 8 h. The samples were prepared as described in Methods and analyzed using confocal laser scanning microscope (CSLM, Leica, Germany). Scale bar—10 µm.

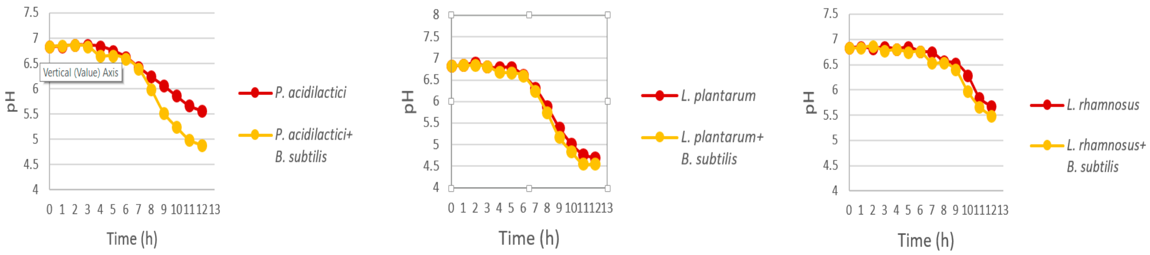

(**a**) (**b**) (**c**)

Figure S2. The effect of growth in dual-species biofilm on the medium acidification rate by LAB cells. Changes in pH of the MMRS medium during growth of the LAB cells as mono culture or in co-culture with *B. subtilis*. Red line represents changes in the pH of the medium by the single-species culture of LAB, while a yellow line presents the ΔpH of the medium following incubation of *L. plantarum* (A), *P. acidilactici* (B) and *L. rhamnosus* (C) in co-culture with *B. subtilis*.

Table S1. Bacterial strains used in this study.

	Strain	Genotype	Strain description	Reference
Bacillus subtilis	**NCIB3610**	WT	Laboratory strain	[50]
Bacillus subtilis	**YC189**	P_{tapA}-*cfp* in 3610, $Spec^R$	Produces cyan fluorescent protein (CFP) under the control of *tapA* promoter	[51]
Lactobacillus plantarum	NCIBM 12422	WT	Laboratory strain	Lallemand France
Lactobacillus rhamnosus GG	ATCC 53103	WT	Laboratory strain	Isolated from probiotic sample, Swanson probiotics
Pediococcus acidilactici	HK01	WT	New milk-isolate	Dairy farm of Ortal (Israel)
Staphylococcus aureus	ATCC 25923	WT	Laboratory strain	Sela lab collection (ARO, Israel)

Table S2. Primers used for RT-PCR analyses.

Gene	sequence (5′-3′)
16S-F	5' GCGAAGTGCGGGTGATTT 3'
16S-R	5' GCAGTCTATGTGTGTTTACCGTTACCT 3'
rpoB-F	5' TGCCGGTTACGGTTCTTTTG 3'
rpoB-R	5' TGTCGCTGTTTTCTGTGTTATCTTTAT 3'
srfA-F	5' GCGCGTGGGTATGTCAATCT 3'
srfA-R	5' TTGGTTGTCGATTCTGCCTAAA 3'
fenA-F	5' TTATCCGGGTGAACGCATGT 3'
fenA-R	5' TCTGAGCGCCGCTTCAA 3'
